# Association between Human Plasma Chondroitin Sulfate Isomers and Carotid Atherosclerotic Plaques

**DOI:** 10.1155/2012/281284

**Published:** 2011-12-15

**Authors:** Elisabetta Zinellu, Antonio Junior Lepedda, Antonio Cigliano, Salvatore Pisanu, Angelo Zinellu, Ciriaco Carru, Pietro Paolo Bacciu, Franco Piredda, Anna Guarino, Rita Spirito, Marilena Formato

**Affiliations:** ^1^Dipartimento di Scienze Fisiologiche, Biochimiche e Cellulari, Università delgi Studi di Sassari, 07100 Sassari, Italy; ^2^Dipartimento di Scienze Biomediche, Università delgi Studi di Sassari, 07100 Sassari, Italy; ^3^Servizio di Chirurgia Vascolare, Clinica Chirurgica Generale, Università delgi Studi di Sassari, 07100 Sassari, Italy; ^4^Centro Cardiologico “F. Monzino,” IRCCS, Università delgi Studi di Milano, 20122 Milano, Italy

## Abstract

Several studies have evidenced variations in plasma glycosaminoglycans content in physiological and pathological conditions. In normal human plasma GAGs are present mainly as undersulfated chondroitin sulfate (CS). The aim of the present study was to evaluate possible correlations between plasma CS level/structure and the presence/typology of carotid atherosclerotic lesion. Plasma CS was purified from 46 control subjects and 47 patients undergoing carotid endarterectomy showing either a soft or a hard plaque. The concentration and structural characteristics of plasma CS were assessed by capillary electrophoresis of constituent unsaturated fluorophore-labeled disaccharides. Results showed that the concentration of total CS isomers was increased by 21.4% (*P* < 0.01) in plasma of patients, due to a significant increase of undersulfated CS. Consequently, in patients the plasma CS charge density was significantly reduced with respect to that of controls. After sorting for plaque typology, we found that patients with soft plaques and those with hard ones differently contribute to the observed changes. In plasma from patients with soft plaques, the increase in CS content was not associated with modifications of its sulfation pattern. On the contrary, the presence of hard plaques was associated with CS sulfation pattern modifications in presence of quite normal total CS isomers levels. These results suggest that the plasma CS content and structure could be related to the presence and the typology of atherosclerotic plaque and could provide a useful diagnostic tool, as well as information on the molecular mechanisms responsible for plaque instability.

## 1. Introduction

Atherosclerosis is a progressive disease characterized by the accumulation of lipids and fibrous elements in medium and large arteries. Plaque rupture and thrombosis are the most important clinical complication in the pathogenesis of acute coronary syndromes and peripheral vascular disease [1, 2]. Although numerous risk factors such as hypertension, diabetes, and hyperlipidemia are thought to play a role in the development and progression of this pathology [3], the mechanisms underlying plaque formation and progression are still largely unknown. Abnormal expression and structural modifications of arterial chondroitin sulfate proteoglycans (CS-PGs) have been implicated in atherosclerosis progression [4–6]. Arterial CS-PGs are markedly increased in early atherosclerotic lesions, participating in lipid retention, modification, and accumulation. Furthermore, CS-PGs play a key role in inflammation processes associated with atherosclerosis [6]. Numerous *in situ* lines of evidence indicate that plaque instability is caused by a substantial increase of both proteolytic activity and inflammatory state [7]. However, nowadays specific systemic markers for early diagnosis and prognosis of atheromatous lesions have not yet been identified.

Several studies have evidenced variations in plasma glycosaminoglycan (GAG) levels associated with both physiological and pathological conditions, such as strong physical training [8], chronic lymphocytic leukaemia and essential thrombocythaemia [9], lupus erythematosus [10], and mucopolysaccharidosis [11, 12].

Chondroitin sulfate (CS) is the main GAG in normal human plasma. It consists of 12–18 repeating disaccharide units, each containing a hexuronic acid linked by *β* (1→3) bond to a *N*-acetyl-D-galactosamine residue, of which about 30% is sulfated at C-4 hydroxyl group of the hexosamine. It circulates covalently linked to the proteoglycan bikunin [13], a light subunit carrying the antiproteinase activity of plasma serine-proteinase inter-*α*-inhibitor (I*α*I). Several data suggest that bikunin plays an important role in inflammation. The I*α*I family molecules are synthesized in hepatocytes where one or two polypeptides, called the heavy chains (HCs), are linked to the CS chain of bikunin. After a stimulus, the I*α*I molecules leave the circulation and, in extravascular sites, the HCs are transferred from CS chain to the locally synthesized hyaluronic acid (HA) to form the serum-derived hyaluronan-associated-protein- (SHAP-) HA complex, which plays important roles in stabilizing extracellular matrices and it is often associated with inflammatory conditions [14]. Moreover, it has been described that bikunin also acts as a growth factor for endothelial cells, regulates the intracellular calcium levels, and inhibits kidney stone formation and smooth muscle cell contraction [15].

The aim of the present study was to evaluate possible correlations between plasma chondroitin sulfate (CS) level/structure and the presence/typology of carotid atherosclerotic lesion.

## 2. Materials and Methods

### 2.1. Sample Collection

CS isomers analyses were conducted on preoperative plasma samples from 47 patients (stenosis > 70%, 70.4 ± 7.9 years old) undergoing carotid endarterectomy, showing either a soft (26 patients) or a hard (21 patients) plaque, and from 46 healthy normolipidemic volunteers (control group), aged from 20 to 75 (47.8 ± 14.4 years old). The main clinical parameters of the patients under study are reported in Table 1. Lipid profiles and homocysteine levels in patients and controls are reported in Table 2. Plaque typology was assessed by ultrasonography using a Mylab 70 Xvision ecocolordoppler equipped with a LA332 AppleProbe 11–3 MHz (Esaote). Plaques were classified according to the Gray-Weale classification [16] in soft, with hypoechoic features (types 1 and 2), and hard, with hyperechoic features (types 3, 4, and 5). Informed consent was obtained before enrolment. The study was approved by the local ethical committees in accordance with institution guidelines.

### 2.2. Plasma CS Isomers Analysis

GAG purification was performed by a microanalytic preparative method, as previously described [17]. Briefly, 500 *μ*L of plasma samples were subjected to proteolytic treatment with papain. Plasma CS was purified by anion exchange chromatography and precipitated with 5 volumes of ethanol at −20°C for 24 h. Subsequently, purified plasma CS isomers were subjected to depolymerization by using chondroitin ABC lyase (0.1 U per 100 *μ*g hexuronic acid) and the unsaturated disaccharides were derivatized with 0.1 mol/L 2-aminoacridone (AMAC) [18].

Separation and quantitation of chondroitin sulfate-derived disaccharides was obtained by capillary electrophoresis (CE) analysis by using a P/ACE capillary electrophoresis system (Beckman) equipped with a 75 mm id and 47 cm length uncoated fused-silica capillary using a 60 mmol/L sodium acetate buffer, containing 0.05% methylcellulose [17]. Separations were carried out at 25°C and monitored with a laser-induced fluorescence (LIF) detector at 488 nm excitation and 520 nm emission wavelengths. For quantitative analyses a homemade standard CS was purified from human plasma pools by a preparative approach. Briefly, standard CS isomers were obtained from papain-treated plasma by anion exchange chromatography, assayed for hexuronic acid content [19], lyophilized into aliquots, and stored at −20°C. For Δ-disaccharide analyses, a calibration curve was determined by submitting plasma-purified CS isomers to chondroitin ABC lyase treatment and derivatization procedure.

CS levels were expressed as *μ*g of hexuronic acid per mL of plasma (*μ*g_UA_/mL), and CS charge density was evaluated as ratio of 4-sulfated Δ-disaccharides (ΔDi-4S) and total unsaturated disaccharides (ΔDi-4S + ΔDi-nonS).

### 2.3. Statistical Analyses

Statistical analyses were conducted by using SigmaStat software (Systat Software, Inc.). Student's *t*-test was used for evaluating differences between normally distributed data, while the Mann-Whitney Rank Sum test was applied to nonparametric ones. Values of *P* < 0.05 were considered to be significant. Correlations between CS content, CS charge density, and age were determined by the Pearson Product Moment Correlation test with 95% confidence intervals.

## 3. Results

Plasma samples from atherosclerotic patients and from healthy volunteers were analysed. Patients were sorted in two groups according to the typology of their carotid lesion (either soft or hard) as evaluated by ecocolordoppler ultrasonography. The two groups of patients did not differ for the main clinical (Table 1) and biochemical parameters (Table 2), neither as a whole nor subsorting for gender. Lipid profiles and homocysteine levels (Table 2) were similar in patient and control groups (*P* > 0.05).

CE was used to analyze the quantity and fine structure of plasma CS isomers. The adopted method allows the simultaneous determination of hyaluronan- and CS-derived disaccharides with a good reproducibility of both the migration times (CV%, 0.25) and the peak areas (CV%, 1.4). Intra- and interassay CVs were 5.37 and 7.23%, respectively, and analytical recovery was about 86% [17]. This method has been validated by a comparison with a reference assay (fluorophore-assisted carbohydrate gel electrophoresis) using the Passing and Bablock regression analysis and the Bland-Altman test as specific statistical methods for measurement comparison [17].

CE analyses of plasma CS isomers showed the presence of two main unsaturated disaccharides, the nonsulfated (ΔDi-nonS) and the 4-monosulfated (ΔDi-4S).

To rule out any influence of age on our analytical and structural results, we evaluated its association with both content and charge density of plasma CS in control group. In this respect, no correlation was found even after sorting controls for gender. The age did not correlate neither with ΔDi-nonS (*r* = −0.219; *P* = 0.143) nor with ΔDi-4S (*r* = −0.002; *P* = 0.143) levels.

Statistical analyses allowed us to evidence significant differences between the whole group of patients and the group of controls (Table 3) consisting in an increase of undersulfated CS levels (4.46 ± 1.59 versus 3.56 ± 0.99 *μ*g_UA_/mL, *P* = 0.002), with consequent increase of total CS content (6.28 ± 2.28 versus 5.17 ± 1.48 *μ*g_UA_/mL, *P* = 0.009) and reduction in its charge density (28.6 ± 4.6 versus 30.8 ± 4.5%, *P* = 0.022). Interestingly, after sorting for plaque typology, we evidenced significant differences in total CS concentration only in patients with a soft plaque with respect to healthy subjects (6.76 ± 2.16 versus 5.17 ± 1.48 *μ*g_UA_/mL, *P* < 0.001), due to significantly higher levels of both ΔDi-nonS (4.73 ± 1.48 versus 3.56 ± 0.99 *μ*g_UA_/mL, *P* = 0.001) and ΔDi-4S (2.03 ± 0.75 versus 1.61 ± 0.56, *P* = 0.017), whereas patients with hard plaques showed quite normal levels. In plasma from patients with a soft plaque both normosulfated and undersulfated CS isomers were significantly increased and their relative proportions were unchanged. So, no significant changes in CS charge density were detected. On the contrary, in plasma from patients with hard plaques, although the levels of both CS isomers were quite normal, a significant difference in their relative proportions was found with respect to controls, producing a significantly lower CS charge density (−12%).

No differences by Student's *t*-test in both total CS content and CS charge density emerged after subsorting both patients and controls for gender.

## 4. Discussion

Human normal plasma contains principally an undersulfated form of chondroitin at a concentration of about 4 *μ*g_UA_/mL [20], whose origin and physiological roles have not yet been fully elucidated. It circulates covalently linked to bikunin [13, 14], or as a main product of tissue catabolism or produced by blood cells, such as lymphocytes, associated with a variety of plasma proteins [21, 22]. It is known that the plasma GAG association with low-density lipoproteins could affect some of their physicochemical properties [23] and that some physiological and pathological conditions could lead to an increase in plasma GAG levels [8–12]. Moreover, mediators of inflammation such as some cytokines and growth factors are able to modulate the size, the degree, and the pattern of sulfation, as well as the degree of epimerization of the GAG chains [24–26].

In this work we studied possible correlations between plasma CS level/structure and presence/typology of carotid atherosclerotic lesion. In a previous paper [17], we developed a sensitive and reproducible analytical method for the quantitative and structural evaluation of human plasma CS isomers using capillary electrophoresis (CE). Herein, this analytical approach was adopted to evaluate CS concentrations and structural characteristics in 46 healthy human subjects and in 47 atherosclerotic patients having either a soft or a hard plaque.

Plaque typology was assessed by duplex ultrasonography which represents a noninvasive method for the carotid plaque characterization. This technique allows to detect areas with different shades of grey that provide information on plaque consistency. In particular, hypoechoic features are associated with lipid-rich carotid plaques, while hyperechoic features are with fibrous or fibrocalcific ones [16]. In this respect, several recent ultrasound studies have demonstrated that hypoechoic plaques, with a low GSM (gray-scale median) value, were associated with an increased risk of cerebrovascular ischemic events [27–29]. So, the soft plaque shows characteristics of instability and propension to rupture. It is generally held that plaque instability is caused by a substantial increase in proteolytic activity and inflammatory status.

To rule out any influence of age on our results, we evaluated its association with plasma CS content and sulfation pattern by means of Pearson's correlation. In this respect, no correlation was found. Few conflicting data regarding the relationship between plasma GAG content and ageing are present in the literature. Some investigations found that total plasma GAG content does not vary with age [30, 31]. Conversely, a positive correlation between total plasma GAG levels and age has been reported in males [32]. Qualitative analysis of intact plasma GAGs by using cellulose acetate electrophoresis has shown a decline of CS with proceeding age [33], while the structural analysis of plasma CS after depolymerization with specific chondro-/dermatolyases has revealed a significant increase of CS amount and its charge density depending on age [34]. On the basis of our results, both plasma CS levels and its charge density seem to be unaffected by age. Moreover, no correlation was found with gender.

With regard to the influence of atherosclerotic lesion presence, we found significant differences in total plasma CS content (+21.4%), between the group of patients and the controls. These differences were ascribable to significantly higher levels of undersulfated CS in these patients (+25.3%).

The statistical analyses of the influence of atherosclerotic lesion typology on plasma CS content and sulfation pattern showed that in presence of soft plaques CS content was significantly increased, without significant changes in its charge density, whereas in presence of hard plaques its charge density was reduced, without changes in its content.

The significance of the observed modifications in plasma CS isomers of patients undergoing endarterectomy could be related to different PG/GAG metabolism in vascular tissue in presence of soft/hard atherosclerotic plaques. Atherosclerosis has been associated with a biosynthetic imbalance of chondroitin sulfate proteoglycans [4–6]. It has been described that the ratio of 6-sulfated to 4-sulfated disaccharides is increased in atherosclerotic type II aortas and significantly decreased in atherosclerotic type V, indicating that vascular concentration of GAGs is differently affected during the progression of the disease [4]. Increased levels of both undersulfated and normosulfated CS have been described in postoperative serum samples of patients submitted to coronary artery bypass surgery and proposed as indicative of an inflammatory state of the patient [35].

In this regard, it has been reported that in inflammatory diseases the CS chains carried by bikunin increase in size proportionally to the severity of the inflammatory response [36], while their sulfation degree decreases [37].

Moreover, several studies reported that sulfation pattern of CS isomers may be important for their protective role from oxidative damage [38–43]. The antioxidant properties of GAGs could be explained by both GAGs chelating properties on divalent cations (such as Cu^2+^ and Fe^2+^, responsible for the initiation of hydroxyl radical reactions) and their improving effects on endogenous antioxidant defences. In particular, the anti-oxidant properties of CS isomers seem to be related to the sulfation at position 4 of galactosamine residue of disaccharide units.

On a whole, our data show that plasma CS level and structure are significantly different in atherosclerotic patients with respect to controls and that these differences do not depend on age. Moreover, the obtained results suggest that both content and distribution of the circulating CS isomers are associated with the presence as well as with the typology of carotid plaque. If plasma CS levels and structure reflect, almost partly, the CS-PGs metabolism in affected vascular tissues, the determination of plasma CS isomers may provide information on molecular mechanisms of atherosclerosis progression.

Therefore, evaluating content and distribution of plasma CS isomers could be useful in diagnosis of carotid atherosclerosis. Further studies on subjects with preclinical atherosclerosis would be advisable. Indeed, finding an association between these molecules and early atherosclerosis stages could provide an important tool for the follow-up of patients in attempting to prevent inauspicious cerebrovascular events.

## Figures and Tables

**Table 1:** Main clinical parameters of the 47 patients undergoing carotid endarterectomy according to plaque typology.

	Hard (21)	Soft (26)	All (47)
Age	70.9 ± 5.1	70.2 ± 9.3	70.4 ± 7.9
Sex ratio (m/f)	2/1	1.3/1	1.6/1
Body mass index (kg/m^2^)	26.9 ± 3.4	27.9 ± 3.8	27.5 ± 3.6
Symptomatic (%)	44.4	56.2	51
Transient ischemic attack (%)	33.3	25	28
Ictus (%)	11.1	31.2	22
Diabetes (%)	44.4	25	34
On therapy (%)	75	75	75
HbA_1C_ (%)	4.5 ± 2.6	4.5 ± 3.0	4.5 ± 2.8
Hypertension (%)	100	93.75	97
On therapy (%)	100	100	100
Dyslipidemic (%)	77.8	75	76
On therapy (%)	100	100	100
Smokers (%)	77.8	68.7	73.0
CRP (mg/dL)	1.16 ± 1.05	1.34 ± 0.84	1.26 ± 0.92

**Table 2:** Lipid profiles and homocysteine levels in patients and controls.

	Hard (21)	Soft (26)	All patients (47)	Controls (46)
Age	70.9 ± 5.1	70.2 ± 9.3	70.4 ± 7.9	47.8 ± 14.4
Sex ratio (m/f)	2/1	1.3/1	1.6/1	1/1.5
Triglycerides (mg/dL)	119.9 ± 55.1	112.7 ± 67.1	115.4 ± 61.7	86.6 ± 28.7
Total cholesterol (mg/dL)	187.9 ± 43.3	164.5 ± 38.3	173.2 ± 41.0	175.7 ± 17.5
LDL cholesterol (mg/dL)	102.4 ± 35.9	90.9 ± 35.6	95.2 ± 35.4	108.3 ± 12.6
HDL cholesterol (mg/dL)	61.2 ± 17.0	51.5 ± 10.9	55.1 ± 14.0	50.1 ± 10.9
Homocysteine (*μ*mol/L)	12.5 ± 4.7	11.5 ± 4.1	11.8 ± 4.3	10.32 ± 3.27

**Table 3:** Total plasma CS, ΔDi-4S, and ΔDi-nonS levels and CS charge density in control subjects, in the totality of patients and according to plaque typology.

	Total CS (*μ*g_UA_/mL)	ΔDi-4S (*μ*g_UA_/mL)	ΔDi-nonS (*μ*g_UA_/mL)	^#^CS charge density (%)
Controls (*n* = 46)	5.17 ± 1.48	1.61 ± 0.56	3.56 ± 0.99	30.8 ± 4.5
Patients (*n* = 47)	6.28 ± 2.28	1.82 ± 0.77	4.46 ± 1.59	28.6 ± 4.6
Soft (*n* = 26)	6.76 ± 2.16	2.03 ± 0.75	4.73 ± 1.48	29.7 ± 4.0
Hard (*n* = 21)	5.69 ± 2.34	1.57 ± 0.74	4.12 ± 1.69	27.1 ± 4.8
Patient versus control	0.009*	0.132	**0.002**	**0.022**
Soft versus control	**<0.001**	**0.017**	**0.001**	0.337
Hard versus control	0.321*	0.81	0.169	**0.004**
Hard versus soft	0.114	**0.04**	0.202	0.055

*P* values obtained by Student's *t*-tests for normally distributed parameters or by *the Mann-Whitney Rank Sum tests for nonparametric ones are reported. ^#^CS charge density was evaluated as ratio between ΔDi-4S and the sum of ΔDi-nonS and ΔDi-4S. Significant differences are reported in bold (*P* < 0.05).
